# Retention of health and care workers needs more than listening to needs: towards an active role in governance and a comprehensive implementation strategy

**DOI:** 10.1093/eurpub/ckag186

**Published:** 2026-10-13

**Authors:** Ellen Kuhlmann, André Beja, Monica Brinzac, Alicja Domagala, Martijn Felder, Ber Oomen, Erica Richardson, Tomas Zapata, Tiago Correia

**Affiliations:** Institute for Economics, Labour and Culture, Goethe-University Frankfurt, Frankfurt, Germany; WHO Collaborating Center on Health Workforce Policies and Planning, Instituto de Higiene e Medicina Tropical, Universidade Nova de Lisboa, Lisbon, Portugal; WHO Collaborating Center on Health Workforce Policies and Planning, Instituto de Higiene e Medicina Tropical, Universidade Nova de Lisboa, Lisbon, Portugal; Global Health and Tropical Medicine, GHTM, Associate Laboratory in Translation and Innovation Towards Global Health, LA-REAL, Instituto de Higiene e Medicina Tropical, IHMT, Universidade Nova de Lisboa, Lisbon, Portugal; Department of Public Health, Faculty of Political, Administrative and Communication Sciences, Babeș-Bolyai University, Cluj-Napoca, Romania; European Public Health Association (EUPHA), Utrecht, The Netherlands; Institute of Public Health, Jagiellonian University Medical College, Krakow, Poland; Erasmus School of Health Policy and Management, Erasmus University Rotterdam, Rotterdam, The Netherlands; European Specialist Nurses Organisation (ESNO), Arnhem, The Netherlands; European Observatory on Health Systems and Policies, London, United Kingdom; World Health Organisation Regional Office for Europe, Copenhagen, Denmark; WHO Collaborating Center on Health Workforce Policies and Planning, Instituto de Higiene e Medicina Tropical, Universidade Nova de Lisboa, Lisbon, Portugal; Global Health and Tropical Medicine, GHTM, Associate Laboratory in Translation and Innovation Towards Global Health, LA-REAL, Instituto de Higiene e Medicina Tropical, IHMT, Universidade Nova de Lisboa, Lisbon, Portugal

## Introduction

Poor retention is a key driver of the health and care workforce crisis with direct effects on service provision, quality of care, migration flows, health system resilience, and trust in governments. The root causes are complex but well known, stretching beyond demographic change. Poor working conditions and severe mental health pressures are prominent in the debate, while persistent gender inequalities, weak integration of migrant health and care workers, limited career prospects, and growing violence against frontline workers are equally relevant [1–3].

There is no shortage of policy recommendations on how to respond. The WHO framework for action for the European region calls on Member States to improve working conditions, ensure fair remuneration, promote gender equality, protect mental and physical health, prevent abuse and violence, support ethical recruitment, and address retention in rural and underserved areas [1]. The EU has added to these efforts through specific investment in the nursing profession, including the Nursing Action initiative, and through renewed attention to safe staffing levels and health workforce performance assessment [4–6]. Research has also generated evidence on planning, forecasting, migration, professional development, staffing, primary care, and implementation capacities for change [7–11].

Yet policy efforts have not responded effectively to the needs of health and care workers. In many countries, retention is likely to deteriorate further, worsening shortages and weakening preparedness. The problem is not only a lack of policy commitments or evidence; it is an implementation and governance failure. Retention policies often name what should be done, but say less about how change should be organized, who has authority to act, and how workers themselves can shape the conditions that determine whether they remain in the workforce.

In this Viewpoint, we argue that listening to health and care workers is necessary but insufficient. Retention must move from a workforce management problem to a health system governance concern. This requires implementation capacity, participatory governance, and an active role for health and care workers as co-governors of system change, not merely as the objects of retention policy.

## What capacities can support retention governance?

Retention governance begins from a simple premise: capacities for change already exist across the workforce and across health systems, but they are often underused. Students and early-career professionals are one example. Mentoring, financial support, and clear career paths can support retention, but younger professionals also bring expectations and competencies that are relevant to future systems, including prevention, leadership, and systems thinking [12–14]. They should not only be treated as a group at risk of leaving, but as a constituency able to inform health systems transformation. The same applies to older workers. Retaining experienced staff may require more flexible work arrangements, adapted tasks and opportunities to transfer knowledge. These are not marginal human resource measures. They are part of a broader strategy to protect institutional memory, sustain mentoring capacity and maintain continuity of care in systems already operating under pressure.

Nurses, as the largest workforce group and as frontline professionals, are especially exposed to workload, stress, unsafe staffing, and violence. This is why the current European focus on nursing is important, including strategies for retention, safe staffing, and the WHO/Europe Nursing Action initiative [4–6]. However, nurses are not only a vulnerable group requiring protection. They are central actors in redesigning everyday practice, improving team organization and making retention measures credible at workplace level. If retention policies fail nurses, they fail not only workforce stability but also the wider project of building resilient health systems.

## From implementation gaps to a participatory governance architecture

Most European countries have expanded policy activity, addressing pay, mental health, training, migration, staffing, or violence, but they did not invest in implementation and create the governance architecture to connect these actions over time and across levels. Retention measures are often fragmented across ministries, professional bodies, employers, education providers, and local authorities.

Evidence points to several sites of implementation capacity. Governments can strengthen knowledge exchange, cross-border partnerships, and integrated professional education, as small-country experiences demonstrate [15]. Decentralized systems can use stakeholder-driven policy co-creation and legal tools that empower local authorities to adapt retention measures to local realities [16]. Organizations can invest in middle managers, who translate national policies into everyday working conditions and who often determine whether reforms are experienced as support or as another administrative burden [17].

At the micro-level, health and care workers can identify problems that formal indicators miss and can test workable solutions in real settings. But this requires more than consultation; workers need spaces where they can influence priorities before decisions are finalized. Participation must be institutionalized across agenda-setting, design, implementation, monitoring, and accountability.

This is the point of moving towards the governance of retention and health and care workforce-driven transformation, where workers are recognized as persons, professionals, and system actors rather than as an infrastructure to be managed [8]. Retention governance differs from retention policy. Policy sets objectives; governance creates the conditions through which objectives can be implemented, contested, corrected, and sustained. Retention, therefore, needs a comprehensive participatory governance architecture and a coherent European strategy connected to national, regional, organizational, and professional arenas where implementation actually happens [2, 7, 10].

## Conclusions and policy directions

Improving retention still requires better working conditions, mental health protection, fair remuneration, safety, gender equality, ethical recruitment, safe staffing, and career development. These priorities remain essential, yet they are unlikely to succeed if treated as isolated interventions or renewed promises added to systems that do not sufficiently care for their health and care workforce and that many workers no longer trust. Retention becomes credible when health and care workers can see that policy commitments change the organization of work and that they have a recognized role in shaping that change.

Three shifts are therefore needed. First, retention must move from a workforce shortage problem to a condition of health system resilience and security. Second, retention must move from policy recommendations to implementation architecture, linking European strategy with national, regional, local, and organizational governance. Third, health and care workers must move from being objects of retention policy to active players in system change.

## Data Availability

No original data were collected for this article.
